# Supplementary material for: A Likelihood Approach for Real-Time Calibration of Stochastic Compartmental Epidemic Models
Source: PLoS Comput Biol. 2017 Jan 17;13(1):e1005257. doi: 10.1371/journal.pcbi.1005257 (PMC5240920; doi:10.1371/journal.pcbi.1005257)
Supplement: S1 File — (TAR.GZ) [file pcbi.1005257.s014.tar.gz › HSPH_Online-SI-Revision/PF08/n10-extreme/n10-extreme_PF_table-8.pdf]

1	R0	{2.68692, 12.5884}	{1.93473, 16.3534}	{{1.2265, 10.7139}, {2.65059, 20.}}	{30.0595, 36.6717}	inf	21.	201.273	{70.4402, 365.793}	858.441	0.892305	newly	13.	109.49	{31., 210.}	742.692	0.820066	Reff	2.6759	1.84276	{1.18026, 2.44836}	32.1911	ar	9089.	7236.22	{4050., 9232.}	21.6099
2	R0	{1.79802, 12.5412}	{1.77922, 18.7551}	{{1.5083, 14.9899}, {2.07379, 20.}}	{10.233, 49.6898}	inf	125.	385.894	{259.173, 528.313}	208.715	0.472335	newly	89.	161.7	{105., 233.}	81.7303	0.240036	Reff	1.74857	1.6532	{1.41419, 1.86629}	9.81881	ar	7412.	7831.07	{6606., 8827.}	11.0028
3	R0	{2.6174, 10.1105}	{2.00543, 13.5521}	{{1.1546, 8.23415}, {2.86001, 18.8819}}	{26.3983, 44.3212}	inf	4.	145.659	{8.16113, 315.841}	3544.17	1.32099	newly	3.	101.	{7., 207.}	3272.	1.31149	Reff	2.61609	1.90754	{1.14204, 2.64568}	28.6307	ar	9109.	7019.93	{2558., 9257.}	23.4914
4	R0	{2.27532, 14.3149}	{1.70968, 18.4166}	{{1.44704, 14.3361}, {2.07547, 20.}}	{25.257, 29.9505}	inf	174.	386.867	{267.802, 520.099}	122.337	0.330676	newly	107.	166.27	{109., 234.}	57.1308	0.179018	Reff	2.20251	1.58591	{1.34284, 1.89838}	28.0983	ar	8478.	7521.15	{6474., 8736.}	12.9881
5	R0	{2.96649, 18.0674}	{1.97479, 17.9503}	{{1.41323, 13.3967}, {2.69003, 20.}}	{33.5883, 12.2167}	inf	107.	272.113	{137.149, 524.059}	154.311	0.347507	newly	64.	131.87	{56., 253.}	111.234	0.271874	Reff	2.92051	1.8779	{1.35562, 2.55724}	35.6996	ar	9476.	7789.56	{5618., 9660.}	18.5739
6	R0	{2.94778, 16.0002}	{1.99299, 16.1049}	{{1.25141, 10.7106}, {2.79492, 20.}}	{32.6303, 20.581}	inf	56.	213.778	{77.5532, 383.727}	281.789	0.494517	newly	41.	120.21	{37., 250.}	197.829	0.388788	Reff	2.92125	1.88248	{1.22206, 2.47222}	35.5615	ar	9321.	7449.75	{4072., 9428.}	20.6161
7	R0	{2.17562, 11.7184}	{1.97542, 14.9933}	{{1.26508, 7.53422}, {2.96869, 20.}}	{23.9568, 40.4797}	inf	23.	178.746	{68.6787, 330.514}	677.158	0.807294	newly	18.	120.82	{35., 245.}	571.222	0.702907	Reff	2.16844	1.88728	{1.19452, 2.75622}	24.5516	ar	8345.	7276.91	{4334., 9563.}	20.414
8	R0	{2.7904, 17.8966}	{1.84168, 17.9295}	{{1.42747, 13.6368}, {2.37468, 20.}}	{34.1253, 12.6892}	inf	98.	287.326	{154.947, 483.071}	193.189	0.428381	newly	46.	135.47	{68., 231.}	194.5	0.417747	Reff	2.74575	1.72322	{1.36621, 2.17262}	37.2404	ar	9285.	7555.48	{5877., 9116.}	18.985
9	R0	{2.51574, 16.376}	{1.80841, 17.6998}	{{1.39191, 12.5487}, {2.30113, 20.}}	{28.7064, 18.07}	inf	112.	281.687	{156.47, 453.466}	151.506	0.362901	newly	55.	131.38	{70., 226.}	139.2	0.336323	Reff	2.47171	1.70111	{1.32827, 2.21991}	31.2014	ar	8937.	7438.47	{5692., 9079.}	17.9207
10	R0	{2.56405, 18.1787}	{1.82269, 16.9994}	{{1.42946, 12.1093}, {2.18907, 20.}}	{29.8091, 15.154}	inf	124.	301.752	{183.05, 448.539}	143.349	0.351479	newly	69.	150.84	{74., 256.}	119.362	0.294035	Reff	2.51226	1.70232	{1.348, 2.07099}	32.5035	ar	9005.	7531.58	{5812., 8860.}	17.0807
11	R0	{2.475, 17.1709}	{1.59068, 19.3456}	{{1.50621, 18.6721}, {1.69859, 20.}}	{35.73, 14.5024}	inf	144.	461.378	{383.909, 551.655}	220.401	0.499495	newly	77.	179.6	{141., 221.}	133.247	0.358183	Reff	2.41461	1.4466	{1.37745, 1.55883}	40.0896	ar	8870.	7353.13	{6862., 7884.}	17.1068
12	R0	{2.14974, 11.8619}	{1.78727, 17.1592}	{{1.38081, 11.8766}, {2.29991, 20.}}	{20.741, 47.9062}	inf	45.	265.562	{161.604, 386.191}	490.137	0.742113	newly	37.	128.71	{70., 212.}	247.865	0.506114	Reff	2.13147	1.69273	{1.31807, 2.18596}	22.7905	ar	8414.	7356.29	{5526., 9013.}	16.2072
13	R0	{2.20385, 14.4767}	{1.61188, 18.8565}	{{1.46809, 14.1801}, {1.88178, 20.}}	{26.8608, 31.7746}	inf	171.	426.071	{323.03, 595.365}	149.164	0.384941	newly	105.	172.57	{124., 241.}	64.3524	0.202006	Reff	2.13685	1.45222	{1.32417, 1.66362}	32.0393	ar	8347.	7100.65	{6194., 8311.}	15.5044
14	R0	{2.35671, 13.8357}	{1.9026, 16.9534}	{{1.40034, 12.0401}, {2.55389, 20.}}	{23.355, 28.3892}	inf	81.	263.433	{142.417, 395.779}	225.226	0.472431	newly	53.	134.64	{63., 207.}	155.057	0.356168	Reff	2.32631	1.80881	{1.33406, 2.4301}	24.6175	ar	8719.	7694.48	{5707., 9383.}	15.1059
15	R0	{1.91368, 10.1082}	{1.67325, 17.9989}	{{1.4297, 13.5785}, {2.05403, 20.}}	{15.3979, 78.12}	inf	100.	357.659	{240.818, 476.468}	257.659	0.536008	newly	95.	157.29	{102., 220.}	66.5158	0.199983	Reff	1.87579	1.55469	{1.37692, 1.85747}	17.481	ar	7486.	7310.35	{6191., 8664.}	10.9373
16	R0	{2.48727, 14.882}	{1.93886, 17.1503}	{{1.44967, 11.7101}, {2.57616, 20.}}	{24.5467, 23.7406}	inf	30.	258.445	{139.848, 420.734}	761.484	0.893772	newly	19.	133.06	{52., 218.}	600.316	0.778632	Reff	2.47508	1.84317	{1.37862, 2.43068}	26.7732	ar	8894.	7835.82	{6091., 9434.}	14.2998
17	R0	{2.46795, 13.1915}	{2.1255, 14.6691}	{{1.39342, 9.02584}, {3., 20.}}	{24.0895, 27.8314}	inf	43.	208.092	{68.5634, 405.488}	384.512	0.596088	newly	22.	146.14	{36., 296.}	566.545	0.705185	Reff	2.45487	2.03108	{1.37303, 2.87088}	24.555	ar	8796.	7851.65	{5303., 9615.}	16.0715
18	R0	{2.96603, 16.7543}	{1.92579, 16.9739}	{{1.39747, 11.1275}, {2.55447, 20.}}	{35.1403, 18.7949}	inf	59.	273.91	{140.913, 492.754}	364.254	0.609922	newly	37.	140.36	{62., 252.}	279.351	0.512283	Reff	2.93429	1.82531	{1.34328, 2.45665}	37.7939	ar	9394.	7734.46	{5642., 9468.}	18.1705
19	R0	{2.25216, 9.90465}	{2.028, 15.0297}	{{1.37797, 9.21932}, {2.91272, 20.}}	{22.2257, 56.2687}	inf	7.	203.53	{65.9919, 402.679}	2807.57	1.36961	newly	5.	132.37	{30., 272.}	2547.4	1.29483	Reff	2.24946	1.92519	{1.32759, 2.60937}	22.3188	ar	8631.	7657.34	{5202., 9468.}	15.9866
20	R0	{2.70837, 18.1923}	{1.76006, 17.2472}	{{1.43261, 12.2638}, {2.2719, 20.}}	{35.0139, 14.3475}	inf	85.	252.229	{164.718, 400.731}	196.74	0.441759	newly	45.	121.95	{68., 194.}	171.	0.394899	Reff	2.67506	1.66918	{1.34919, 2.13656}	37.602	ar	9183.	7284.65	{5688., 9074.}	20.9824
21	R0	{2.30288, 15.5877}	{1.66921, 19.014}	{{1.48602, 16.2601}, {1.88078, 20.}}	{27.5164, 23.9767}	inf	145.	390.984	{273.17, 496.375}	169.644	0.418361	newly	76.	159.36	{106., 216.}	109.684	0.306786	Reff	2.2437	1.53709	{1.36744, 1.72026}	31.4931	ar	8674.	7384.73	{6381., 8375.}	15.0757
22	R0	{2.82208, 10.7544}	{1.99493, 13.4509}	{{1.1546, 6.93514}, {2.86001, 18.8819}}	{30.0362, 38.6627}	inf	4.	142.698	{8.16113, 315.841}	3470.15	1.30633	newly	9.	99.18	{7., 206.}	1014.44	0.895874	Reff	2.82039	1.89753	{1.14204, 2.64568}	32.8596	ar	9333.	6973.24	{2558., 9257.}	25.3132
23	R0	{1.69923, 10.1366}	{1.76974, 17.8915}	{{1.40313, 13.4604}, {2.21733, 20.}}	{15.7093, 76.5517}	inf	114.	315.712	{182.819, 504.242}	176.941	0.408595	newly	88.	142.53	{75., 224.}	68.1932	0.200528	Reff	1.65454	1.65395	{1.301, 2.05087}	14.6758	ar	7055.	7383.59	{5880., 8944.}	14.9529
24	R0	{2.64944, 19.054}	{1.68224, 18.1294}	{{1.43934, 13.3453}, {2.06072, 20.}}	{36.5056, 10.2306}	inf	333.	418.994	{278.767, 553.648}	32.2472	0.113034	newly	143.	180.06	{118., 242.}	33.6643	0.1197	Reff	2.50266	1.52163	{1.33747, 1.77629}	39.1993	ar	9147.	7371.2	{6163., 8672.}	19.4604
25	R0	{2.53191, 10.9214}	{1.77356, 17.5812}	{{1.38664, 12.857}, {2.19452, 20.}}	{30.667, 62.3274}	inf	6.	258.742	{150.526, 396.981}	4212.37	1.6024	newly	9.	120.77	{65., 209.}	1241.89	1.08973	Reff	2.52811	1.67849	{1.32729, 2.10498}	33.7743	ar	9009.	7255.31	{5574., 9002.}	20.4661
26	R0	{1.89155, 6.76944}	{2.07347, 14.8369}	{{1.25576, 8.99584}, {3., 20.}}	{26.1118, 120.483}	inf	21.	215.245	{66.997, 457.464}	924.975	0.912759	newly	28.	146.18	{35., 301.}	424.5	0.597589	Reff	1.88512	1.97311	{1.22379, 2.78953}	24.279	ar	7348.	7645.58	{4521., 9577.}	21.6971
27	R0	{2.63149, 15.6979}	{1.9549, 17.3794}	{{1.44337, 13.119}, {2.60173, 20.}}	{26.7644, 18.9397}	inf	43.	256.975	{147.426, 361.025}	497.615	0.740696	newly	23.	128.76	{54., 207.}	459.826	0.695716	Reff	2.61465	1.86619	{1.38311, 2.51512}	29.0046	ar	9104.	7890.04	{6126., 9436.}	14.808
28	R0	{2.85141, 12.6992}	{1.96636, 13.5989}	{{1.15409, 6.93514}, {2.86095, 18.8819}}	{31.6133, 28.064}	inf	11.	155.218	{10.489, 318.363}	1321.27	0.96649	newly	9.	116.54	{9., 207.}	1206.44	0.926614	Reff	2.84685	1.86693	{1.12509, 2.71024}	34.4936	ar	9317.	6833.71	{2426., 9275.}	26.704
29	R0	{2.54244, 12.9085}	{1.72286, 18.3081}	{{1.44037, 15.0267}, {2.10482, 20.}}	{32.3081, 42.5589}	inf	35.	282.06	{179.766, 432.897}	705.885	0.881906	newly	22.	124.37	{79., 189.}	465.318	0.722516	Reff	2.52846	1.61982	{1.35308, 1.91716}	35.9363	ar	9034.	7208.28	{5944., 8655.}	20.5643
30	R0	{2.10982, 11.7636}	{1.74391, 17.762}	{{1.41026, 11.9172}, {2.16586, 20.}}	{19.9802, 52.3216}	inf	84.	306.272	{175.807, 492.492}	264.61	0.526785	newly	65.	138.83	{73., 224.}	113.615	0.292664	Reff	2.07501	1.61615	{1.30159, 1.89099}	23.3007	ar	8224.	7236.99	{5540., 8761.}	14.8688
31	R0	{2.76312, 15.5396}	{1.92434, 15.1739}	{{1.24653, 9.95629}, {2.51831, 20.}}	{31.3598, 19.4855}	inf	70.	220.889	{83.8057, 360.106}	217.698	0.435267	newly	46.	131.5	{40., 243.}	193.478	0.410835	Reff	2.73438	1.82214	{1.18123, 2.39973}	33.8366	ar	9220.	7247.88	{4034., 9169.}	22.0011
32	R0	{2.21727, 15.5439}	{1.58411, 19.6415}	{{1.51548, 19.2221}, {1.67327, 20.}}	{28.556, 26.57}	inf	175.	507.695	{424.204, 641.812}	190.111	0.456868	newly	111.	193.79	{152., 242.}	74.5856	0.234945	Reff	2.14809	1.4036	{1.33299, 1.49561}	34.6581	ar	8359.	7321.36	{6879., 7880.}	12.5343
33	R0	{2.72912, 10.8159}	{1.96702, 15.6511}	{{1.24416, 10.7008}, {3., 20.}}	{30.5757, 48.3109}	inf	10.	199.991	{76.6671, 338.08}	1899.91	1.22826	newly	16.	116.55	{37., 232.}	628.438	0.775664	Reff	2.72393	1.88184	{1.19785, 2.84744}	32.3742	ar	9196.	7293.77	{4034., 9525.}	21.9296
34	R0	{2.20928, 10.1308}	{1.95018, 12.8441}	{{1.1315, 5.43578}, {2.79685, 18.8819}}	{24.3113, 44.765}	inf	22.	138.951	{8.16113, 313.302}	551.09	0.720814	newly	14.	141.5	{9., 206.}	926.714	0.788995	Reff	2.20155	1.8547	{1.06165, 2.6297}	24.5995	ar	8388.	6628.43	{1756., 9199.}	25.672
35	R0	{2.10103, 15.2673}	{1.7119, 16.7761}	{{1.38069, 11.6898}, {2.13058, 20.}}	{20.8226, 23.3281}	inf	193.	311.161	{192.651, 508.257}	62.8364	0.185643	newly	113.	150.51	{86., 234.}	44.5044	0.149885	Reff	2.038	1.58673	{1.2905, 1.97948}	23.3699	ar	8264.	7076.59	{5543., 8750.}	17.05
36	R0	{2.74914, 19.2775}	{1.67622, 19.1519}	{{1.46201, 18.063}, {2.05571, 20.}}	{39.0274, 5.6912}	inf	143.	381.967	{278.755, 544.144}	167.109	0.415139	newly	67.	155.73	{108., 214.}	132.433	0.35083	Reff	2.68618	1.54451	{1.37426, 1.77938}	42.5017	ar	9301.	7388.87	{6421., 8582.}	20.5583
37	R0	{2.78161, 16.0136}	{1.81491, 17.5312}	{{1.38847, 12.8646}, {2.20024, 20.}}	{34.7531, 19.8494}	inf	38.	255.648	{148.972, 397.38}	572.758	0.796081	newly	25.	121.14	{68., 195.}	384.56	0.648487	Reff	2.76659	1.7085	{1.34904, 2.12258}	38.2452	ar	9240.	7436.11	{5503., 8933.}	19.846
38	R0	{2.4454, 8.33501}	{2.01849, 15.098}	{{1.37797, 9.21932}, {2.80213, 20.}}	{23.6739, 82.7318}	inf	3.	198.143	{64.0702, 402.679}	6504.78	1.7219	newly	9.	128.6	{30., 256.}	1328.89	1.02235	Reff	2.44369	1.91775	{1.32702, 2.57552}	24.6591	ar	8853.	7658.73	{5202., 9456.}	16.3135
39	R0	{2.9154, 12.3861}	{2.07781, 14.4154}	{{1.26007, 7.29508}, {3., 20.}}	{29.6545, 32.4643}	inf	14.	190.771	{69.288, 391.536}	1262.65	1.04523	newly	12.	135.54	{37., 249.}	1029.5	0.929329	Reff	2.91044	1.98729	{1.22556, 2.93878}	32.0981	ar	9371.	7614.17	{4242., 9609.}	19.7956
40	R0	{2.40923, 15.1963}	{1.86713, 17.6743}	{{1.4047, 13.1015}, {2.56532, 20.}}	{24.6693, 23.6788}	inf	149.	309.97	{172.876, 498.766}	108.034	0.28641	newly	86.	145.92	{76., 233.}	74.5814	0.216233	Reff	2.34731	1.74778	{1.32629, 2.38891}	26.5916	ar	8735.	7597.47	{5758., 9398.}	16.1313
41	R0	{2.63137, 15.238}	{1.68818, 17.8261}	{{1.38101, 12.3508}, {2.08622, 20.}}	{35.844, 23.3851}	inf	88.	286.021	{180.405, 472.355}	225.024	0.482457	newly	67.	126.07	{76., 190.}	88.5224	0.244688	Reff	2.59269	1.5759	{1.31437, 1.85753}	39.2176	ar	9150.	7050.76	{5641., 8697.}	23.0017
42	R0	{2.48769, 19.4593}	{1.58348, 19.4625}	{{1.50497, 18.5775}, {1.65812, 20.}}	{36.3473, 2.98995}	inf	353.	515.071	{434.412, 613.874}	45.9123	0.160426	newly	145.	198.44	{165., 237.}	36.8828	0.132037	Reff	2.34714	1.38995	{1.32189, 1.48984}	40.7812	ar	8932.	7261.98	{6831., 7631.}	18.697
43	R0	{2.61199, 10.214}	{2.00543, 13.5521}	{{1.1546, 8.23415}, {2.86001, 18.8819}}	{26.3162, 43.2236}	inf	1.	145.659	{8.16113, 315.841}	14 465.9	1.90883	newly	2.	101.	{7., 207.}	4954.	1.4735	Reff	2.61147	1.90754	{1.14204, 2.64568}	28.5504	ar	9061.	7019.93	{2558., 9257.}	23.2536
44	R0	{2.50994, 18.3382}	{1.60374, 19.4437}	{{1.48908, 18.8838}, {1.77603, 20.}}	{36.1045, 7.95237}	inf	194.	475.718	{377.53, 641.792}	145.215	0.381229	newly	107.	185.44	{146., 241.}	73.3084	0.229373	Reff	2.42761	1.45812	{1.38254, 1.5919}	39.9362	ar	8827.	7413.73	{6766., 8201.}	16.0182
45	R0	{2.44283, 16.8338}	{1.69682, 17.6604}	{{1.37728, 12.8715}, {2.10215, 20.}}	{30.8783, 16.9237}	inf	169.	286.007	{183.993, 418.058}	70.7355	0.207728	newly	105.	130.27	{79., 200.}	37.5143	0.127986	Reff	2.37394	1.58756	{1.30137, 1.86707}	33.1517	ar	8878.	7075.82	{5472., 8497.}	20.8349
46	R0	{2.51815, 18.1462}	{1.68457, 18.7819}	{{1.47617, 14.8828}, {2.05155, 20.}}	{33.1028, 10.7608}	inf	163.	407.782	{274.299, 562.921}	150.173	0.383272	newly	80.	169.72	{107., 234.}	112.15	0.304872	Reff	2.44689	1.53952	{1.37504, 1.79247}	37.0826	ar	8978.	7421.54	{6400., 8701.}	17.3364
47	R0	{2.88885, 16.8987}	{1.67584, 17.3571}	{{1.37348, 12.2525}, {2.14798, 20.}}	{41.9895, 16.2425}	inf	49.	278.497	{166.137, 440.004}	468.361	0.725151	newly	30.	128.65	{73., 201.}	328.833	0.599752	Reff	2.86458	1.56443	{1.29879, 1.86334}	45.387	ar	9337.	6970.97	{5614., 8654.}	25.4057
48	R0	{2.58709, 13.8786}	{1.93465, 15.5527}	{{1.25021, 10.0129}, {2.79542, 20.}}	{28.548, 25.4474}	inf	46.	204.678	{80.9278, 338.08}	344.953	0.577488	newly	26.	121.27	{37., 243.}	366.808	0.580758	Reff	2.57002	1.84343	{1.19141, 2.6213}	30.4742	ar	9146.	7220.28	{4034., 9446.}	22.1085
49	R0	{2.42959, 10.7132}	{2.01849, 15.098}	{{1.37797, 9.21932}, {2.80213, 20.}}	{23.4766, 47.1606}	inf	18.	198.143	{64.0702, 402.679}	1000.8	0.943745	newly	13.	128.6	{30., 256.}	889.231	0.86265	Reff	2.42424	1.91775	{1.32702, 2.57552}	24.3522	ar	8863.	7658.73	{5202., 9456.}	16.3492
50	R0	{2.60513, 13.2796}	{1.93938, 17.0239}	{{1.43836, 12.2656}, {2.59174, 20.}}	{27.1093, 32.506}	inf	24.	261.964	{143.007, 451.489}	991.517	0.991519	newly	29.	130.55	{57., 226.}	350.172	0.599708	Reff	2.59445	1.8432	{1.34956, 2.49569}	29.5698	ar	9095.	7788.32	{5840., 9487.}	16.2949
